# Supplementary material for: Scanning Electron Microscopy and EDX Spectroscopy of Commercial Swabs Used for COVID-19 Lateral Flow Testing
Source: Toxics. 2023 Sep 24;11(10):805. doi: 10.3390/toxics11100805 (PMC10610828; doi:10.3390/toxics11100805)
Supplement: Supplementary file 1 [file toxics-11-00805-s001.zip › toxics-2618287-supplementary.pdf]

Scanning electron microscopy and EDX spectroscopy of commercial swabs used for COVID-19 lateral flow testing (Aparicio-Alonso, M, Torres-Solórzano, V, Méndez Contreras JF, Acevedo-Whitehouse, K)

SUPPLEMENTARY FIGURES

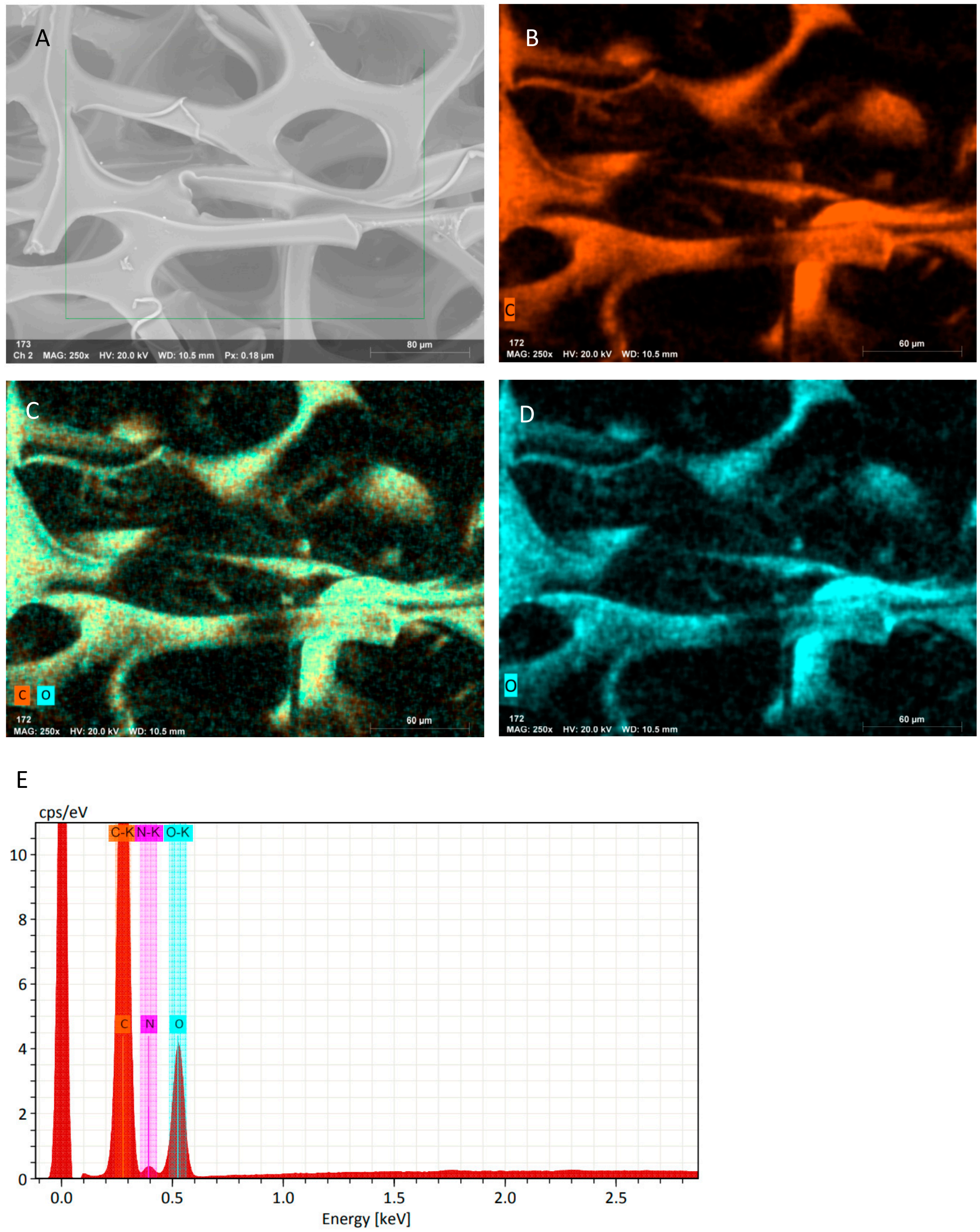

Map

Element	At. No.	Netto	Mass [%]	Mass Norm. [%]	Atom [%]	abs. error [%] (1 sigma)	rel. error [%] (1 sigma)
Carbon	6	338921	57.99	58.27	64.42	6.22	10.73
Nitrogen	7	7919	8.02	8.06	7.64	1.19	14.81
Oxygen	8	85453	33.51	33.67	27.94	3.83	11.44
		Sum	99.52	100.00	100.00		

Figure S1. Analysis of swab 1 (foam head; brand: iHealth). A) Scanning electron micrograph of swab head; B-D) Elemental mapping of elements detected. B: Distribution of Carbon; C: Distribution of Carbon and Oxygen; D; Distribution of Oxygen; E) Chemical elements identified by EDX spectroscopy.

Scanning electron microscopy and EDX spectroscopy of commercial swabs used for COVID-19 lateral flow testing (Aparicio-Alonso, M, Torres-Solórzano, V, Méndez Contreras JF, Acevedo-Whitehouse, K)

SUPPLEMENTARY FIGURES

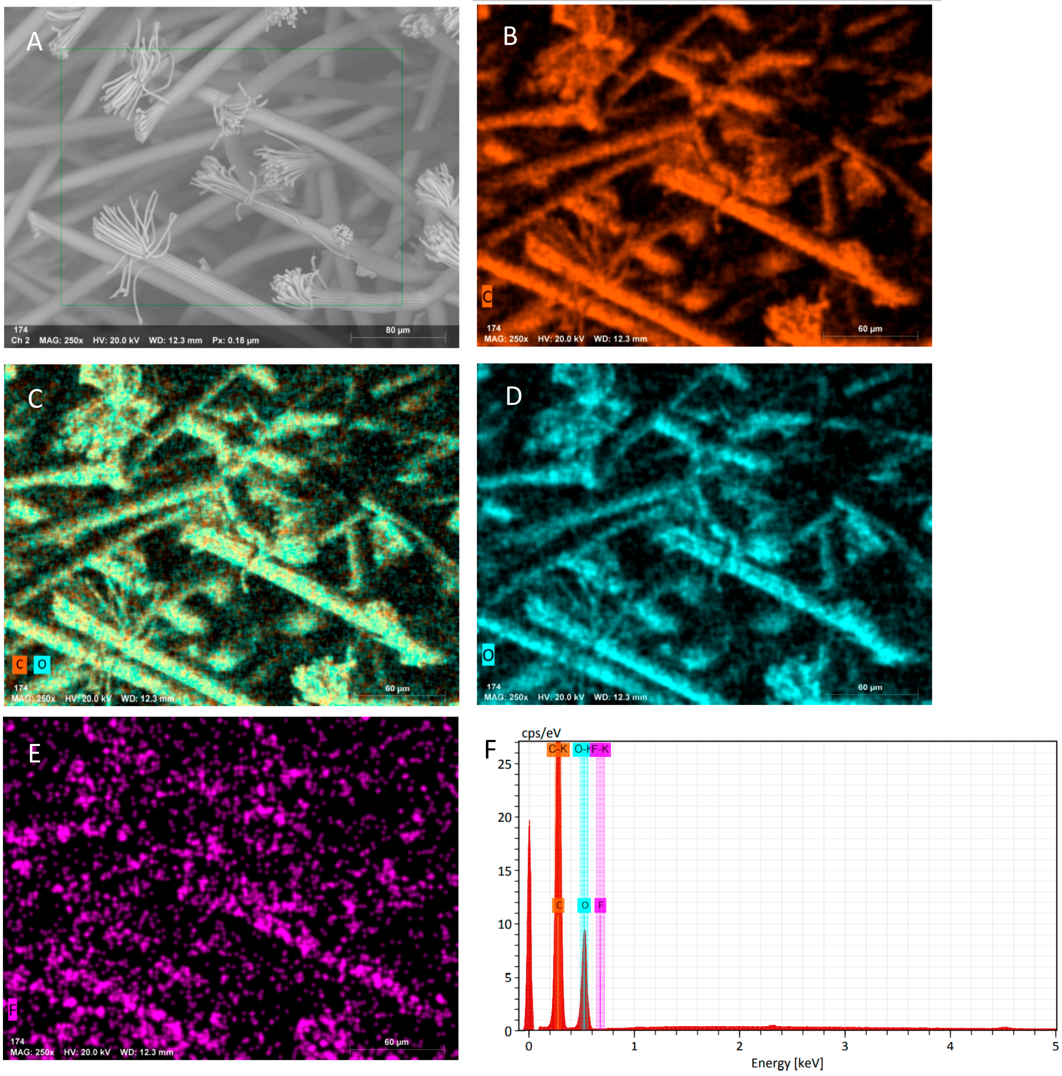

Map

Element	At. No.	Netto	Mass [%]	Mass Norm. [%]	Atom [%]	abs. error [%] (1 sigma)	rel. error [%] (1 sigma)
Carbon	6	392974	62.43	62.66	69.14	6.67	10.68
Oxygen	8	112032	36.69	36.82	30.50	4.13	11.26
Fluorine	9	1665	0.52	0.52	0.37	0.13	24.59
		**Sum**	**99.64**	**100.00**	**100.00**		

Figure S2. Analysis of swab 2 (nylon flocked head; brand: Puritan HydraFlock). A) Scanning electron micrograph of swab head; B-F) Elemental mapping of elements detected. B: Distribution of Carbon; C: Distribution of Carbon and Oxygen; D: Distribution of Oxygen; E: Distribution of Fluorine; F) Chemical elements identified by EDX spectroscopy.

Scanning electron microscopy and EDX spectroscopy of commercial swabs used for COVID-19 lateral flow testing (Aparicio-Alonso, M, Torres-Solórzano, V, Méndez Contreras JF, Acevedo-Whitehouse, K)

SUPPLEMENTARY FIGURES

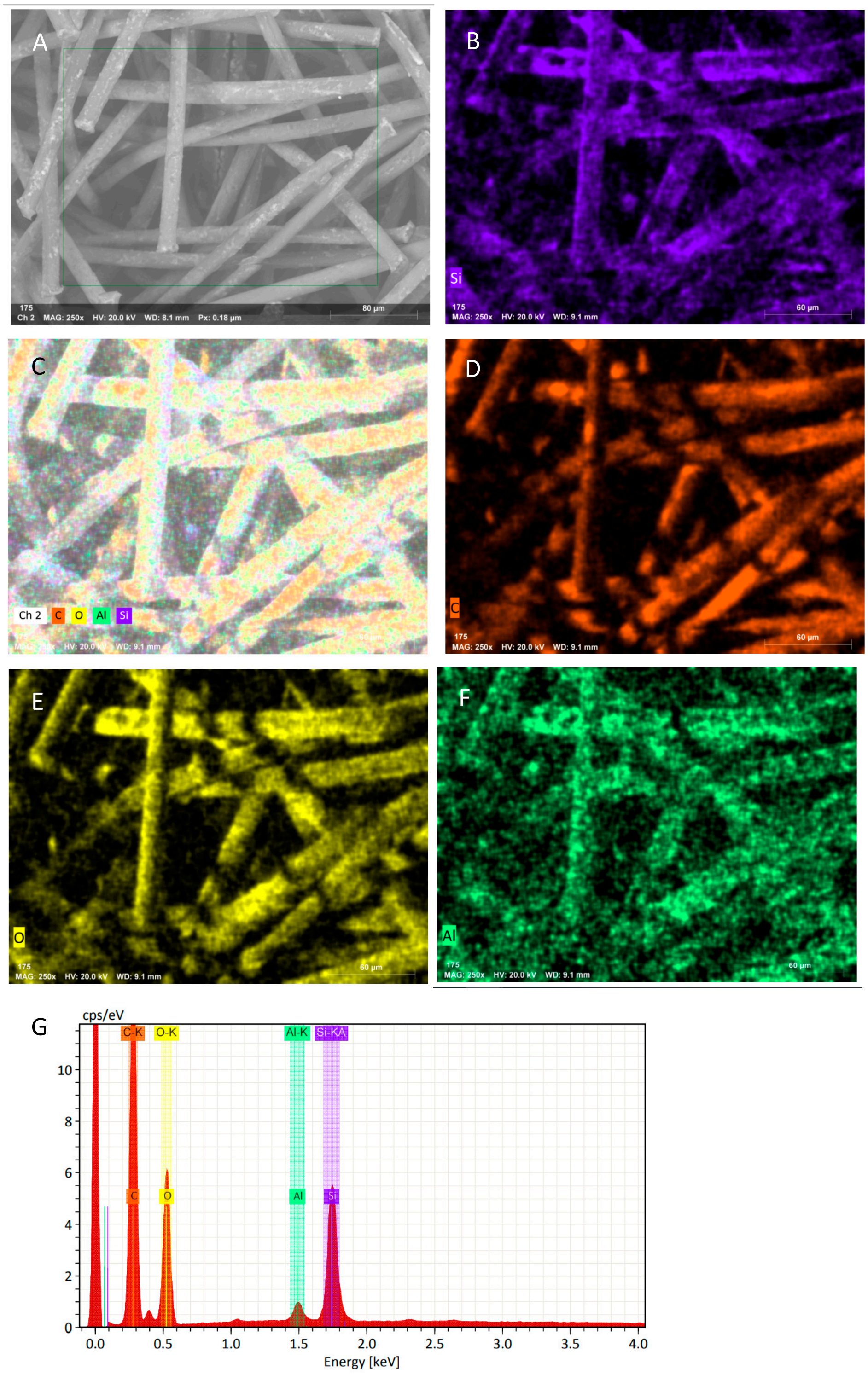

Map

Element	At. No.	Netto	Mass [%]	Mass Norm. [%]	Atom [%]	abs. error [%] (1 sigma)	rel. error [%] (1 sigma)
Carbon	6	207987	53.13	53.49	61.26	5.80	10.92
Nitrogen	7	4714	4.83	4.86	4.78	0.79	16.34
Oxygen	8	96529	36.04	36.29	31.20	4.09	11.36
Fluorine	9	1489	0.53	0.53	0.38	0.13	25.13
Aluminium	13	13154	0.62	0.62	0.32	0.06	9.01
Silicon	14	114757	4.18	4.21	2.06	0.20	4.87
		**Sum**	**99.32**	**100.00**	**100.00**		

Figure S3. Analysis of swab 3 (nylon flocked head; brand: Nasal Swab). A) Scanning electron micrograph of swab head; B-F) Elemental mapping of elements detected. B: Distribution of Silicon; C: Distribution of Carbon, Oxygen, Aluminium, Silicon; D: Distribution of Carbon; E: Distribution of Oxygen; F: Distribution of Aluminium; G) Chemical elements identified by EDX spectroscopy.

Scanning electron microscopy and EDX spectroscopy of commercial swabs used for COVID-19 lateral flow testing (Aparicio-Alonso, M, Torres-Solórzano, V, Méndez Contreras JF, Acevedo-Whitehouse K)

SUPPLEMENTARY FIGURES

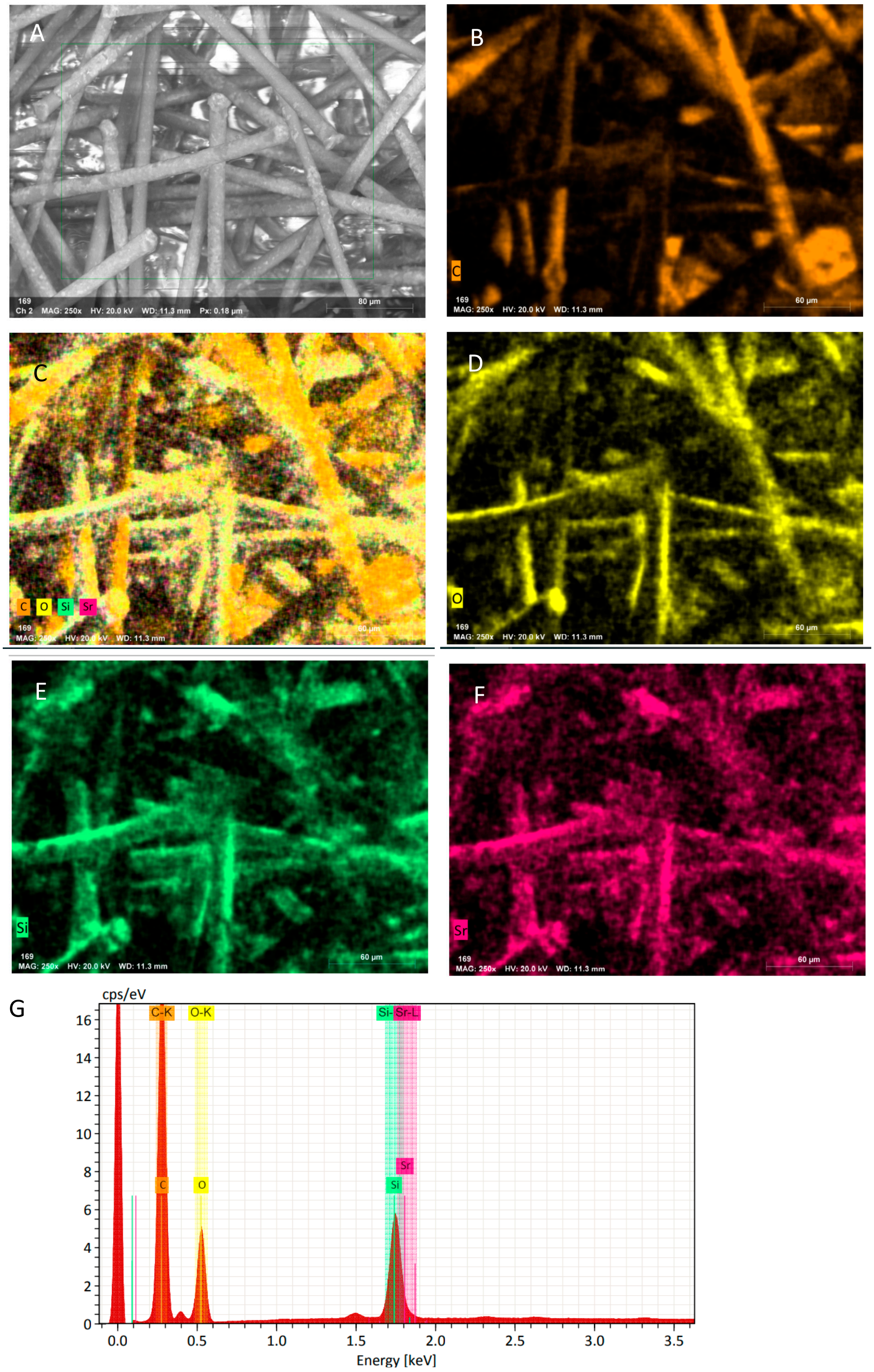

Map

Element	At. No.	Netto	Mass [%]	Mass Norm. [%]	Atom [%]	abs. error [%] (1 sigma)	rel. error [%] (1 sigma)
Carbon	6	329608	59.60	60.19	68.08	6.40	10.74
Nitrogen	7	5644	4.75	4.80	4.66	0.75	15.85
Oxygen	8	93964	29.22	29.51	25.05	3.33	11.39
Silicon	14	146188	3.79	3.83	1.85	0.19	4.93
Titanium	22	18324	0.78	0.79	0.22	0.05	6.09
Strontium	38	16740	0.87	0.88	0.14	0.06	7.11
		**Sum**	**99.02**	**100.00**	**100.00**		

Figure S4. Analysis of swab 4 (nylon head; brand: MANTACC). A) Scanning electron micrograph of swab head; B-F) Elemental mapping of elements detected. B: Distribution of Carbon; C: Distribution of Carbon, Oxygen, Silicon, Strontium; D: Distribution of Oxygen; E: Distribution of Silicon; F: Distribution of Strontium; G) Chemical elements identified by EDX spectroscopy.

Scanning electron microscopy and EDX spectroscopy of commercial swabs used for COVID-19 lateral flow testing (Aparicio-Alonso, M, Torres-Solórzano, V, Méndez Contreras JF, Acevedo-Whitehouse, K)

SUPPLEMENTARY FIGURES

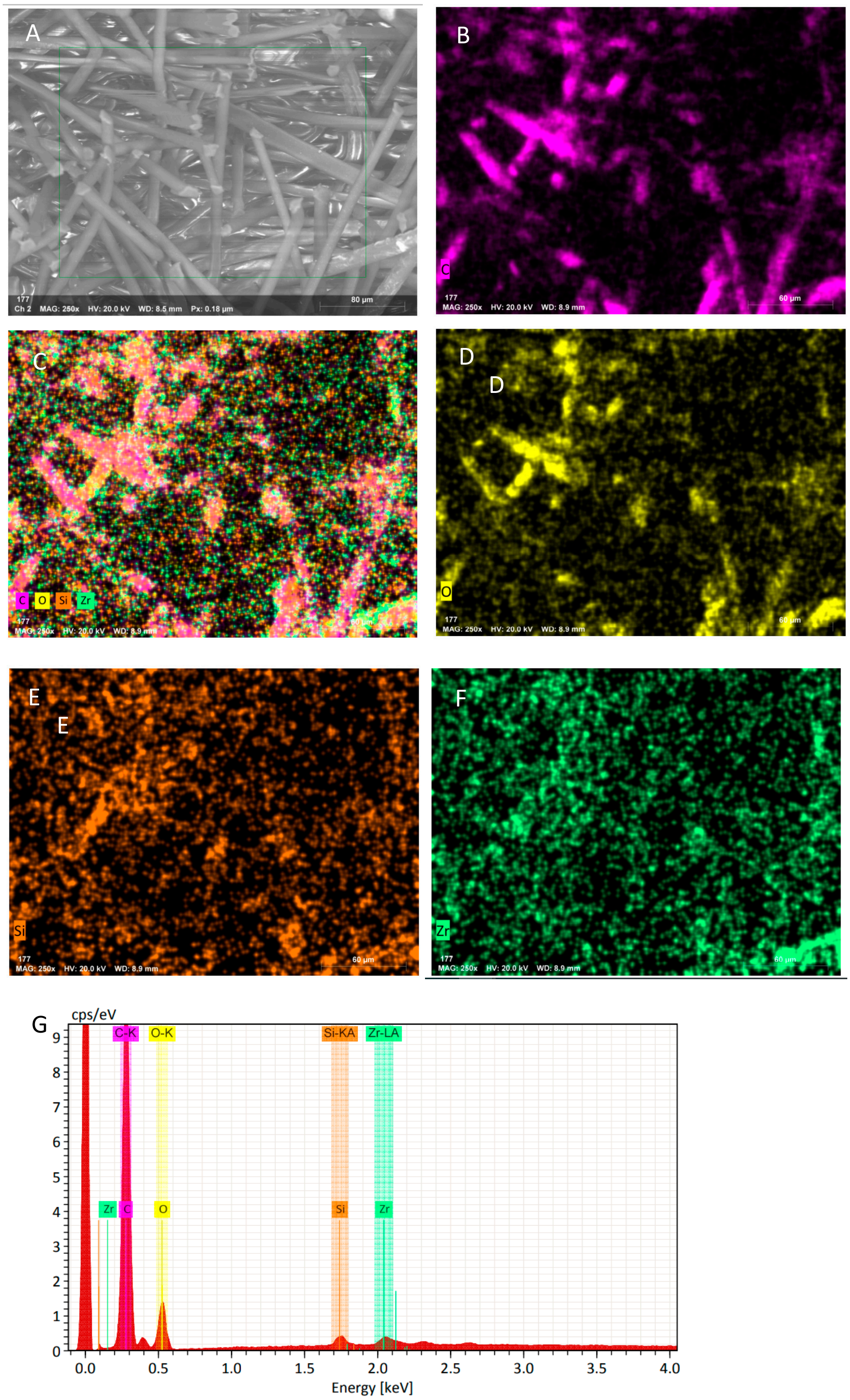

Map

Element	At. No.	Netto	Mass [%]	Mass Norm. [%]	Atom [%]	abs. error [%] (1 sigma)	rel. error [%] (1 sigma)
Carbon	6	95535	63.84	64.04	70.44	7.24	11.33
Nitrogen	7	2488	10.26	10.29	9.70	1.87	18.26
Oxygen	8	13758	23.24	23.31	19.25	3.14	13.52
Silicon	14	3630	0.54	0.55	0.26	0.05	9.44
Titanium	22	3464	0.60	0.60	0.17	0.05	7.55
Zirconium	40	4909	1.21	1.21	0.18	0.08	6.31
		**Sum**	**99.70**	**100.00**	**100.00**		

Figure S5. Analysis of swab 5 (nylon flocked head; brand: FLOQSwabs). A) Scanning electron micrograph of swab head; B-F) Elemental mapping of elements detected. B: Distribution of Carbon; C: Distribution of Carbon, Oxygen, Silicon, Zirconium; D: Distribution of Oxygen; E: Distribution of Silicon; F: Distribution of Zirconium; G) Chemical elements identified by EDX

Scanning electron microscopy and EDX spectroscopy of commercial swabs used for COVID-19 lateral flow testing (Aparicio-Alonso, M, Torres-Solórzano, V, Méndez Contreras JF, Acevedo-Whitehouse, K)

SUPPLEMENTARY FIGURES

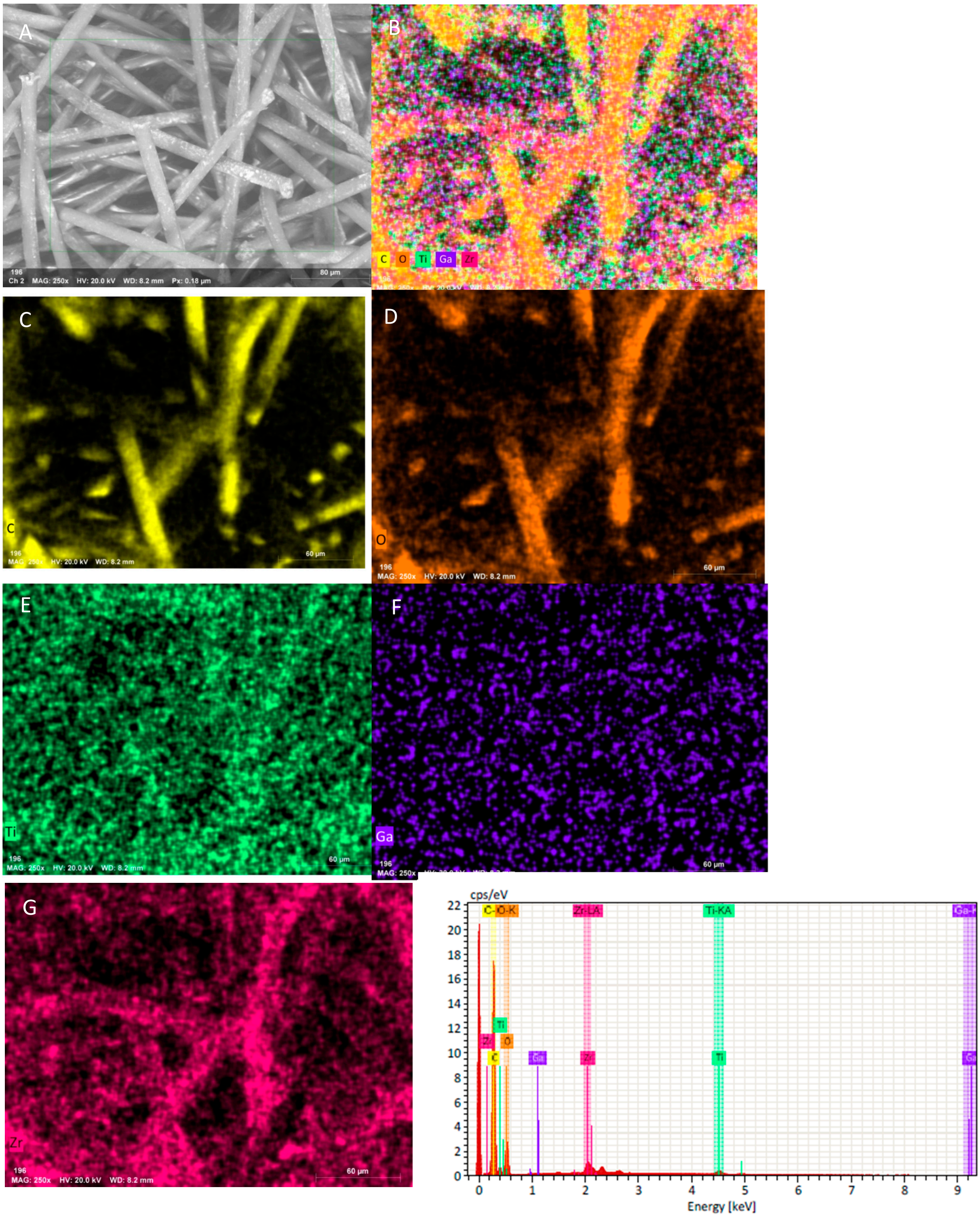

Map

Element	At. No.	Netto	Mass [%]	Mass Norm. [%]	Atom [%]	abs. error [%] (1 sigma)	rel. error [%] (1 sigma)
Carbon	6	297693	61.04	61.45	68.67	6.58	10.77
Nitrogen	7	9118	10.22	10.28	9.85	1.47	14.43
Oxygen	8	53315	24.53	24.69	20.71	2.90	11.83
Sulfur	16	13845	0.54	0.55	0.23	0.05	8.35
Titanium	22	11152	0.65	0.66	0.18	0.04	6.79
Gallium	31	41	0.01	0.01	0.00	0.00	16.54
Zirconium	40	35690	2.35	2.36	0.35	0.12	4.92
		**Sum**	**99.34**	**100.00**	**100.00**		

Figure S6. Analysis of swab 6 (nylon flocked head; brand: Kangdaan). A) Scanning electron micrograph of swab head; B-F) Elemental mapping of elements detected. B: Distribution of Carbon, Oxygen, Titanium, Gallium, and Zirconium; C: Distribution of Carbon; D: Distribution of Oxygen; E: Distribution of Titanium; F: Distribution of Gallium; G: Distribution of Zirconium; H) Chemical elements identified by EDX spectroscopy.

Scanning electron microscopy and EDX spectroscopy of commercial swabs used for COVID-19 lateral flow testing (Aparicio-Alonso, M, Torres-Solórzano, V, Méndez Contreras JF, Acevedo-Whitehouse K)

SUPPLEMENTARY FIGURES

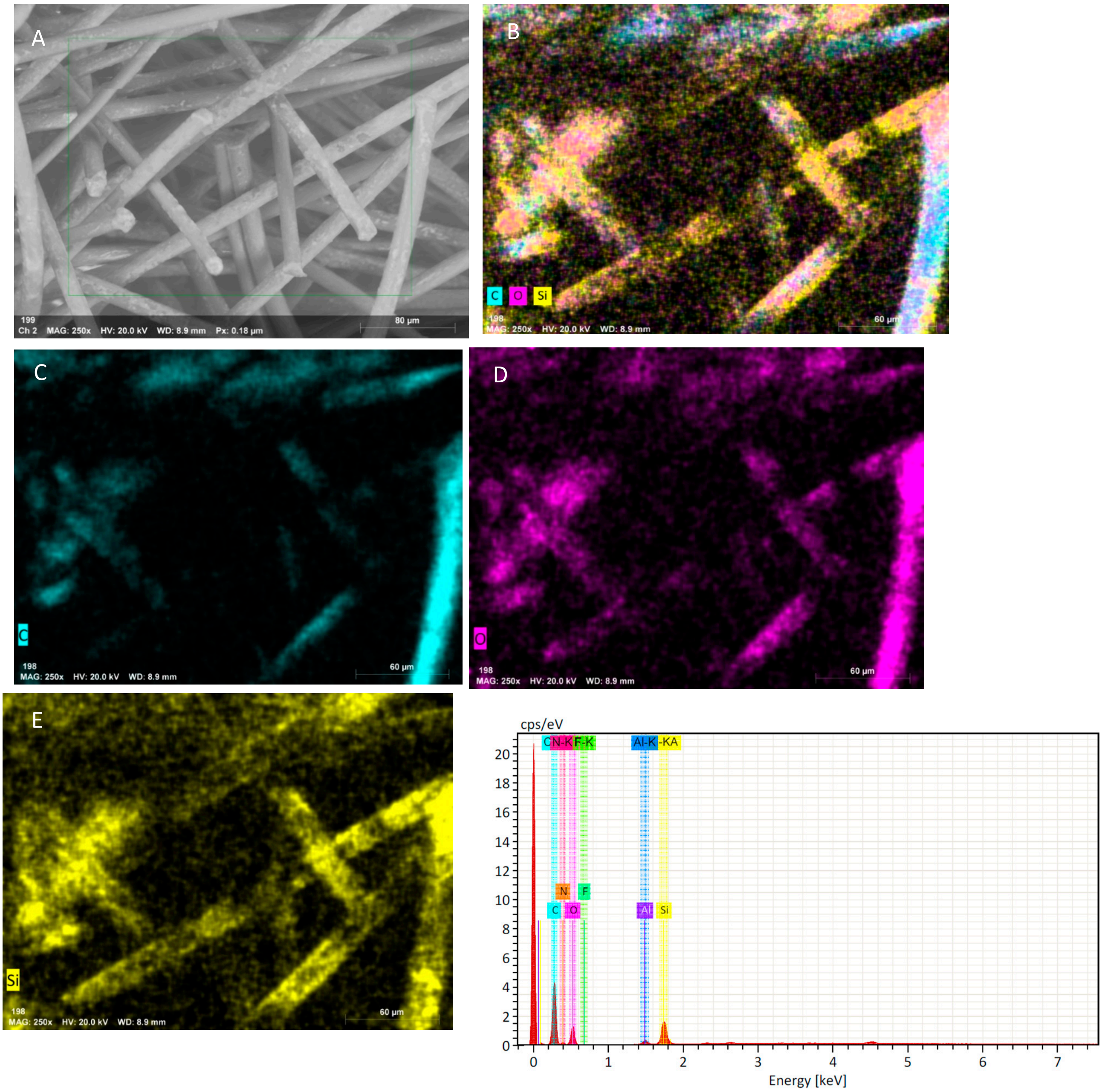

Map

Element	At. No.	Netto	Mass [%]	Mass Norm. [%]	Atom [%]	abs. error [%] (1 sigma)	rel. error [%] (1 sigma)
Carbon	6	74785	56.44	57.01	64.84	6.50	11.51
Nitrogen	7	2092	6.58	6.64	6.48	1.26	19.12
Oxygen	8	24338	29.26	29.55	25.23	3.70	12.65
Fluorine	9	531	0.51	0.52	0.37	0.17	32.21
Aluminium	13	6037	1.14	1.15	0.58	0.08	7.34
Silicon	14	41388	5.07	5.12	2.49	0.24	4.81
		**Sum**	**99.00**	**100.00**	**100.00**		

Figure S7. Analysis of swab 7 (Flocked nylon head; brand: Taizhou). A) Scanning electron micrograph of swab head; B-D) Elemental mapping of elements detected. B: Distribution of Oxygen, Carbon and Silicon; C: Distribution of Carbon; D: Distribution of Oxygen; E: Distribution of Silicon; F) Chemical elements identified by EDX spectroscopy.

Scanning electron microscopy and EDX spectroscopy of commercial swabs used for COVID-19 lateral flow testing (Aparicio-Alonso, M, Torres-Solórzano, V, Méndez Contreras JF, Acevedo-Whitehouse K)

SUPPLEMENTARY FIGURES

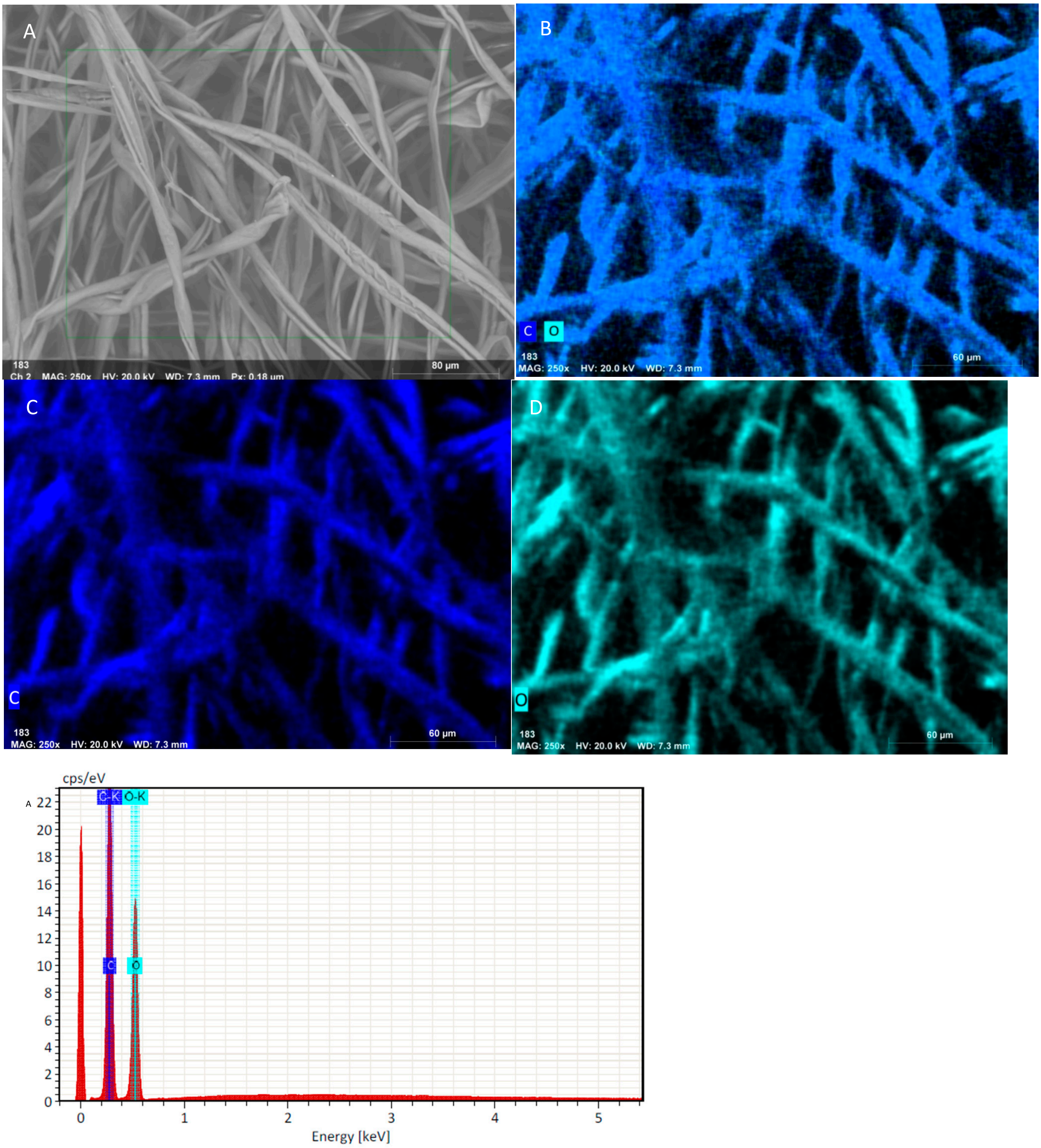

Map

Element	At. No.	Netto	Mass [%]	Mass Norm. [%]	Atom [%]	abs. error [%] (1 sigma)	rel. (1
Carbon	6	250814	48.97	49.08	56.25	5.31	
Oxygen	8	175891	50.31	50.42	43.39	5.54	
Fluorine	9	1316	0.50	0.50	0.36	0.13	
		**Sum**	**99.79**	**100.00**	**100.00**		

Figure S8. Analysis of swab 8 (Cotton head; brand: Generic applicator). A) Scanning electron micrograph of swab head; B-D) Elemental mapping of elements detected. B: Distribution of Oxygen and Carbon; C: Distribution of Carbon; D: Distribution of Oxygen; E) Chemical elements identified by EDX spectroscopy.

Scanning electron microscopy and EDX spectroscopy of commercial swabs used for COVID-19 lateral flow testing (Aparicio-Alonso, M, Torres-Solórzano, V, Méndez Contreras JF, Acevedo-Whitehouse K)

SUPPLEMENTARY FIGURES

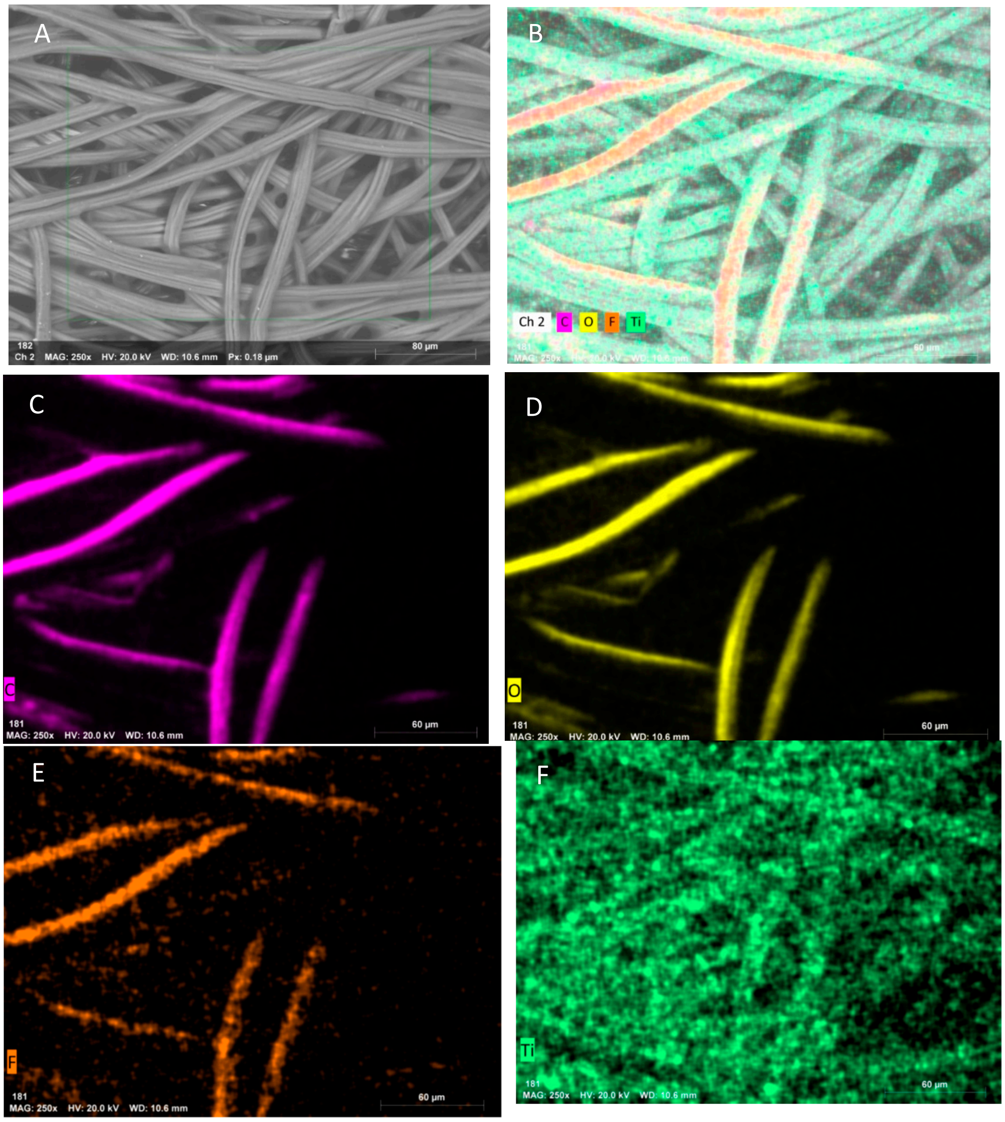

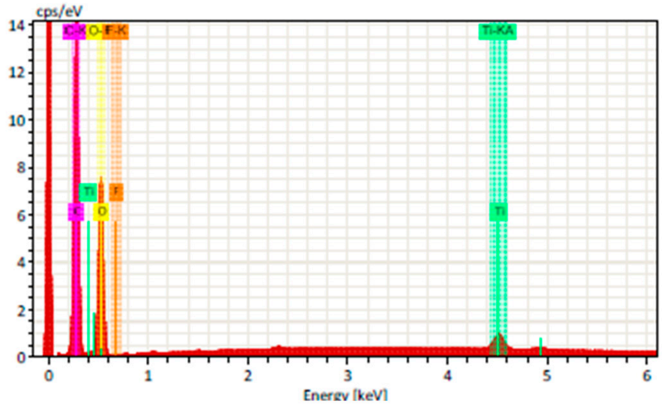

Map

Element	At. No.	Netto	Mass [%]	Mass Norm. [%]	Atom [%]	abs. error [%] (1 sigma)	rel. error [%] (1 sigma)
Carbon	6	458874	50.65	50.99	58.58	5.39	10.64
Oxygen	8	265141	46.78	47.09	40.61	5.07	10.83
Fluorine	9	2706	0.57	0.58	0.42	0.13	22.05
Titanium	22	48044	1.34	1.35	0.39	0.06	4.68
		Sum	99.34	100.00	100.00		

Figure S9. Analysis of swab 9 (cotton head; brand: Transystem). A) Scanning electron micrograph of swab head; B-F) Elemental mapping of elements detected. B: Distribution of Carbon, Oxygen, Fluorine and Titanium; C: Distribution of Carbon; D: Distribution of Oxygen; E: Distribution of Flourine; F: Distribution of Titanium; G) Chemical elements identified by EDX spectroscopy.
